# Retraction: Circulating plant miRNAs can regulate human gene expression *in vitro*

**DOI:** 10.1038/srep46826

**Published:** 2017-05-22

**Authors:** Chiara Pastrello, Mike Tsay, Rosanne McQuaid, Mark Abovsky, Elisa Pasini, Elize Shirdel, Marc Angeli, Tomas Tokar, Joseph Jamnik, Max Kotlyar, Andrea Jurisicova, Joanne Kotsopoulos, Ahmed El-Sohemy, Igor Jurisica

Scientific Reports 6: Article number: 3277310.1038/srep32773; published online: 09 08 2016; updated: 05 22 2017

We are retracting this Article as we no longer have confidence in the data to support our central conclusion – the detection of Brassica oleracea microRNAs in the bloodstream of humans who consumed broccoli.

Upon concerns raised by a reader about the incorrect design of our microRNA primers, we checked the primer sequences and detected antisense design of all the forward primers for broccoli microRNA detection, except for miR824. This invalidates the results described in Figure 1 (except for miR824) and Figure 2 of the paper. We also checked primers designed to detect human genes, and confirmed that all of them were in the correct orientation.

Following the primer analyses, we confirmed by sequencing that the primers for miR824 amplified correct miRNA from broccoli. We then investigated the cause of the trend observed in Figure 2C. We first inspected raw Ct values of miR160, miR2673 and miR21; as visible in Figure 1A below, there was no obvious dose response in any of these PCR products (all p-values shown in Figure 1 come from ANOVA analysis). We then examined raw Ct values of the 3 reference genes (ACTB, B2M and RNU6) that we used for normalization. As shown in Figure 1B, ACTB, B2M and RNU6 were stable (except at T0). While in the paper we calculated expression using geometric mean of reference genes, we also normalized the 3 microRNAs PCRs against the 3 reference genes separately, one at the time. As shown in Figure 1C, B2M is responsible for the trend observed in the paper, most strongly in “T3 160 g” (consumption of 160 g of broccoli per day).

Therefore, we also tested the stability of our 3 reference genes in experiments described in Figure 3 of the paper (transfection of lung cancer cell lines) and Figure 4 (comparison of different treatments in a gastric cell line). We applied ANOVA to raw Ct values of each reference gene, and the resulting p-values are listed in Table 1. All reference genes were stable across lung cancer cell lines, confirming results presented in Figure 3; however, some effect (albeit not significant) was observed on the stability of RNU6 in the gastric cell line when transfected with miR160 or treated with sulphoraphane (Figure 4).

Lastly, we investigated whether repeating our experiments with correct primers targeting broccoli miRNAs could provide evidence for the original finding of this paper. To this aim, we used two protocols: (a) the same protocol used in the published version of the article (with correct and specific forward primers, one set targeting only the microRNA and one set extending to the polyA immediatelynext to the microRNA, listed in Table 2), and (b) a stem-loop protocol^1^. The stem-loop protocol has been described as more efficient in comparison to the ligation method we used originally^2^. We tested two broccoli flower samples, the transfected cell line samples and eight plasma samples from the controlled broccoli feeding trial. Using both protocols we were unable to confirm specific amplification of these miRNAs in human blood. Thus, we were not able to validate the central hypothesis of this paper.

In conclusion, the most appropriate course of action is to retract this paper. We apologize for this error and regret any inconvenience this may have caused. All authors agree to the retraction of the paper.

## Figures and Tables

**Figure 1:**
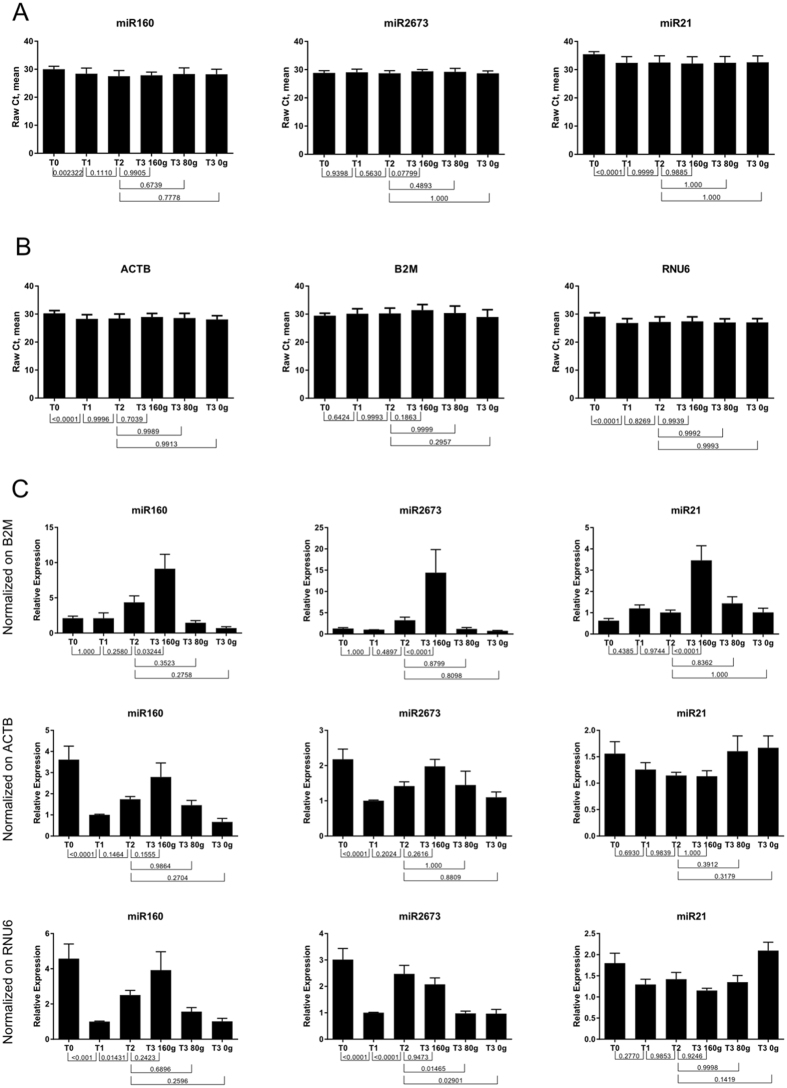
ANOVA comparison of different time points in broccoli feeding trial (Figure 2C in the paper). Shown are comparisons for raw Ct values of microRNAs (**A**), raw Ct values of reference genes (**B**), and (**C**) microRNA values normalized only on one reference gene (listed on the left side of the figure).

**Table 1:** P-values for ANOVA testing across comparisons for each reference gene.

Figure 3			
	miR160 vs AllStar	Siport vs AllStar	NT vs AllStar
ACTB	0.9748	0.9705	0.9977
B2M	0.9887	0.9923	0.9832
RNU6	0.9968	0.9825	0.9997
**Figure 4/Supplementary Table 7**			
	**miR160 vs NT**	**Sulphoraphane vs NT**	**DIM vs NT**
ACTB	0.7293	0.8815	0.6318
B2M	0.9945	0.8172	0.8856
RNU6	0.0792	0.0866	0.9908

**Table 2:** Primers used in the 2 protocols to test validity of our hypothesis.

Mirna	Stem-loop primer	Forward primer
miR160	GTTGGCTCTGGTGCAGGGTCCGAGGTATTCGCACCAGAGCCAACTGGCATACAGG	CCACGTATGCCTGGCTC
miR2673	GTTGGCTCTGGTGCAGGGTCCGAGGTATTCGCACCAGAGCCAACACGAAGAGGAA	CGGCGCGTCTCTTCCTC
miR21	GTTGGCTCTGGTGCAGGGTCCGAGGTATTCGCACCAGAGCCAACTCAACATCAGT	TGCGCGGTAGCTTATCAG
**Qiagen primers**		
**Mirna**	**Primer miRNA specific**	**Primer with A tail**
miR160	TGCCTGGCTCCCTGTATGCCA	TGCCTGGCTCCCTGTATGCCAA
miR2673	TCTCTTCCTCTTCCTCTTCGT	TCTCTTCCTCTTCCTCTTCGTAAA
miR21	TAGCTTATCAGACTGATGTTGA	TAGCTTATCAGACTGATGTTGAA

## References

[b1] Varkonyi-Gasic E. In Methods in molecular biology (Clifton, N.J.) 1456, 163–175 (2017). 10.1007/978-1-4899-7708-3_13 27770365

[b2] Adhikari S. . Hairpin priming is better suited than *in vitro* polyadenylation to generate cDNA for plant miRNA qPCR. Mol. Plant 6, 229–31 (2013). 23024207 10.1093/mp/sss106

